# The Construction of Self in Relationships: Narratives and References to Mental States during Picture-Book Reading Interactions between Mothers and Children

**DOI:** 10.3389/fpsyg.2017.02060

**Published:** 2017-11-28

**Authors:** Dolores Rollo, Emiddia Longobardi, Pietro Spataro, Francesco Sulla

**Affiliations:** ^1^Department of Medicine and Surgery, University of Parma, Parma, Italy; ^2^Department of Dynamic and Clinical Psychology, Sapienza University of Rome, Rome, Italy

**Keywords:** narrative style, mental state language, children, picture-book reading

## Abstract

Previous studies showed that mothers vary in the way in which they discuss past experiences with their children, since they can exhibit *narrative* (*elaborative*) or *paradigmatic* (*repetitive*) styles to different extents. Given this background, the aim of the present study was to analyze differences in the mothers’ use of narrative styles and mental state language (MSL), as a function of children’s age and gender. Thirty dyads consisting of mothers and their 4- to 6-year-old children were observed during a picture-book reading interaction. Maternal utterances were coded according to the categories described by Tessler and Nelson (1994), classifying each mother as *Narrative* or *Paradigmatic*. Eight categories of MSL were analyzed: perceptual, emotional (positive and negative), volitional, cognitive, communicative, and moral. The results confirmed the existence of the two maternal styles observed in the earlier studies. Importantly, we found that the mothers of younger children were more narrative than paradigmatic, whereas the opposite pattern occurred for the mothers of older children (they were more paradigmatic than narrative). As concerns MSL, the results indicated that the use of communicative terms was significantly more frequent for narrative than for paradigmatic mothers, and decreased linearly with children’s age. Lastly, the mothers of younger children referred their MSL more frequently to the book characters than to themselves or to the child. Taken together, these results support the idea that mothers adapt their narrative styles and MSL input to the growing abilities of their children, therefore contributing to the development of social understanding.

## Introduction

Although an implicit sense of self is already apparent before language acquisition (Rochat, 2010), it seems clear that, during infancy, the establishment of an explicit self-concept is primarily driven by the co-occurring growth in linguistic competence. This is because the comprehension and production of grammatical forms such as personal pronouns and verb conjugations require young children to draw an explicit distinction between the roles that can be assumed by themselves or by other people (Bates, 1990). In facts, recent research suggests that the mastery of person reference is closely related to the use of language to express one’s own and others’ mental states (Markova and Smolík, 2014). The underlying assumption is that, to correctly produce personal pronouns and mental state terms, children need to acquire a full awareness of their own desires, feelings, and thoughts (Rochat, 2010); furthermore, they must also understand that other people can have different or even false beliefs about a given situation (Doherty and Perner, 1998; Spataro et al., 2017). Put in other words, they need to acquire an explicit Theory of Mind (ToM) (Flavell, 2004; Wellman and Liu, 2004; Low and Perner, 2012).

Theory of Mind can be defined as the “ability to appreciate the existence of one’s own and others’ mental states (e.g., intentions, emotions, desires), and use them to explain and predict behaviors” (Longobardi et al., 2016b, p. 424; see also Flavell, 2004; Lecce et al., 2015). In the laboratory, this complex set of skills is typically examined by testing children’s understanding of first- and/or second-order false belief (Perner and Wimmer, 1985; Wellman and Liu, 2004). However, a crucial distinction has been drawn between passing false-belief tasks and spontaneously using one’s own ToM abilities during conversations and interactions with peers and adults (Meins et al., 2006; Grazzani and Ornaghi, 2012). Although the existence of a relation between these two domains has been disputed (Tager-Flusberg and Sullivan, 1995; Charman and Shmueli-Goetz, 1998; Meins et al., 2006; Longobardi et al., 2016b), the spontaneous production of mental state language (MSL: a specific type of language including perceptual, physiological, emotional, cognitive and moral terms) has been shown to forerun and predict the acquisition of ToM and, more generally, the emergence of meta-representational awareness (Beeghly et al., 1986; Bartsch and Wellman, 1995). In agreement, significant positive associations between ToM and MSL have been reported by studies examining the behavior of preschool children in a variety of interactive contexts (e.g., book reading; Dunn et al., 1991; Hughes and Dunn, 1998; Rollo and Sulla, 2016).

Importantly for the present purposes, the acquisition of an explicit ToM and the associated production of MSL are strongly influenced by the quality and the frequency of the social interactions to which children are exposed during their first years of life. More specifically, social interactionist approaches to cognitive development posit that the use of language during mother–child conversations has a central importance in promoting the development of mind-reading skills (Ruffman et al., 2002; Symons, 2004; Taumoepeau and Ruffman, 2006, 2008), since children learn to interpret the meaning of their experiences by participating in culturally shared activities (Nelson, 1996; Fivush et al., 2006; Bamberg, 2011). Children’s participation in communicative interactions is also critical to build their ability to construct and share stories with others – a conversational skill that emerges in the early childhood years and is strongly predictive of cognitive, literacy and socioemotional development, as well as school readiness and academic achievement (Deckner et al., 2006; Fivush et al., 2006; Cristofaro and Tamis-LeMonda, 2012). Parents can indeed scaffold the acquisition of children’s narrative ability by sharing personal experiences during family reminiscing, by telling stories during pretend play, or by reading stories from picture books (Whitehurst and Lonigan, 1998; Escobar et al., 2017).

In this broad context, several researchers have begun to examine the social origins of memory, by asking whether differences in maternal narrative styles can affect children’s memory for the narrated events (e.g., Engel, 1986, Unpublished; Fivush, 1994; Nelson, 1996; Nelson and Fivush, 2000; Lange and Carroll, 2003). Fivush et al. (2006), for instance, studied mother–child conversations about past events and found that the children of *elaborative* mothers, who engaged in richly detailed descriptions (e.g., “Memaw and Grandad came over, and Daddy cooked hamburgers out on the grill”: Haden, 1998) and open-ended questions (e.g., “What did we do at the zoo?” and “Who was there with us?”: Fivush et al., 2006), recalled significantly more information than the children of *repetitive* mothers (Fivush and Fromhoff, 1988; Reese et al., 1993; Haden et al., 2001). A similar distinction between narrative and paradigmatic interaction styles has been drawn by Tessler (1986, Unpublished) and Tessler and Nelson (1994), on the basis of previous work by Bruner (1986). According to these authors, *narrative* mothers tend to interpret events and actions in terms of intentions and mental states (e.g., “The frog is jumping on the stone because she’s afraid to drown”: Tessler and Nelson, 1994); in addition, they make frequent references to the autobiographical experiences of their children (e.g., “Do you remember when we went to the rugby match?”: Farrant and Reese, 2000); *paradigmatic* mothers, on the other hand, focus on labeling objects and specifying their relations with superordinate categories (e.g., “What kind of animal is that? A bird, right!”: Tessler and Nelson, 1994), or on repeating the exact content of a previous utterance (e.g., Mother asks, “We had fun, didn’t we?” and in her next turn repeats, “Yes, we had fun”: Haden, 1998). Although all parents produce both narrative and paradigmatic utterances when interacting with their children, most of them show a clear predominance of one style over the other (Lange and Carroll, 2003). Adopting this classification, Tessler and Nelson (1994) coded the spontaneous conversations occurring between mothers and their children during a museum visit: the results showed that the children of narrative mothers recalled the museum objects more accurately than the children of Paradigmatic mothers. Furthermore, in later studies, the use of a narrative style during parent–child interactions has been found to relate to other aspects of children’s cognitive development, including conscience development (Laible and Thompson, 2000), emotion recognition (Ontai and Thompson, 2002), socio-communicative competence (Tadić et al., 2013), and ToM (Welch-Ross, 1997; Ontai and Thompson, 2008).

Besides narrative styles, a large body of research point to the conclusion that the frequency with which mothers refer to MSL during conversations is positively and significantly associated with children’s performance in ToM tasks and the development of their psychological lexicon (Meins et al., 2002; Ruffman et al., 2002; de Rosnay et al., 2004; Slaughter et al., 2007; Ornaghi et al., 2011). Ruffman et al. (2002), for example, found that a composite measure of mothers’ mental state utterances (including the use of desire and think/know terms) at 3 years predicted unique variance in children’s ToM performance at 4 years. Likewise, LaBounty et al. (2008) reported that the frequency of mothers’ emotional explanations at 3.5 years predicted children’s concurrent emotion understanding, whereas the frequency of fathers’ explanations in terms of desires and emotions predicted children’s concurrent and later ToM at 5 years. As concerns the relation between mothers and children’s MSL, two classical studies by Taumoepeau and Ruffman (2006, 2008) showed that maternal references to desires at 15 months significantly predicted children’s later MSL and emotion task performance at 24 months. In addition, mothers’ talk about others’ thought and knowledge to 24-month-old children significantly predicted their MSL at 33 months (see also Furrow et al., 1992; Jenkins et al., 2003; Rudek and Haden, 2005; Symons et al., 2006; Pearson and Pillow, 2016).

### The Present Study

Considering the empirical background briefly summarized in the previous paragraphs, the present study sought to put together these two strands of research by examining the relation between narrative styles (as classified by Tessler and Nelson, 1994 and modified by Rollo, 2003, Unpublished) and the use of MSL in maternal conversations during a shared book reading activity. More specifically, we were interested in determining whether Narrative and Paradigmatic mothers differed in the use of specific categories of mental state terms and/or in the total amount of psychological lexicon directed to children. Although this question has yet to be assessed, the findings illustrated by Welch-Ross (1997) and Ontai and Thompson (2002), showing a direct association between the frequency of elaborative comments in maternal conversations and children’s later ToM, lead to the prediction that elaborative/narrative mothers should produce more mental state terms, compared to repetitive/paradigmatic mothers. This result would be well consistent with previous evidence demonstrating that the mothers’ use of MSL during parent–child interactions promotes ToM in preschool children (Meins et al., 2002; Ruffman et al., 2002; de Rosnay et al., 2004; Symons et al., 2006; Taumoepeau and Ruffman, 2006, 2008; Slaughter et al., 2007; Ornaghi et al., 2011). Hence, the positive effects of maternal elaborations on ToM performance might be potentially mediated by an increased frequency of internal state terms. In addition to this primary aim, we were also focused on determining whether maternal styles varied as a function of children’s age and gender, and whether elaborative/narrative and repetitive/paradigmatic mothers referred their MSL to different agents – the self, the child, the dyad, the context or others (e.g., the characters described in the book). As concerns the first issue, available studies indicate that both mothers and fathers tend to be more elaborative with daughters than with sons (Reese and Fivush, 1993; Reese et al., 1993), and that the mothers of children between 40 and 70 months of age tend to become more elaborative over time. Regarding the second issue, there is at least some evidence showing that maternal references to others’ internal states play a key role in the early development of psychological lexicon. Taumoepeau and Ruffman (2008), for example, found that maternal references to others’ cognitive states at 24 months were the most consistent correlate of children’s later MSL at 33 months. Similarly, a study by Longobardi et al. (2016a) showed that maternal references to others’ mental states at 16 months were associated to the children’s ability to use MSL to communicate their emotions at 20 months.

To summarize, the aims of the present study were: (a) to confirm the validity of the distinction between Narrative and Paradigmatic communicative styles; (b) to investigate whether the mothers’ use of these two communicative styles varied as a function of children’s age; (c) to identify internal state terms in mother–child speech and determine differences related to children’s age and gender; (c) to verify whether mothers classified as Paradigmatic or Narrative differed in their use of MSL; and (d) to investigate whether Narrative and Paradigmatic mothers differed in the relative frequency with which they attributed mental state terms to different agents.

## Materials and Methods

### Participants

Thirty mother–child dyads were observed individually. Only mothers and typically developing children whose first language was Italian were included. Children were 19 females and 11 males ranged in age from 4 years 1 month to 5 years 11 months (*M* = 4.74; *SD* = 0.49). The average age of the mothers was 35 years (*SD* = 3.86).

Children were recruited in a public school of northern Italy; they belonged to a medium socioeconomic group (according to parents’ education and occupation); their receptive language was tested with the Italian adaptation of the Peabody Picture Vocabulary Test-R (PPVT-R; Dunn and Dunn, 1981; Italian version by Stella et al., 2001). The sample had no reported history of psychiatric treatment or neurological disorders.

In order to explore any differences based on children’s age, we created three balanced groups in terms of numerosity and age, each composed of 10 children: (1) average age was 4.05 years (*SD* = 0.04); (2) average age was 5.05 years (*SD* = 0.02); (3) finally, the third group was composed of children who were 5 years and 11 months old.

### Procedure and Measures

Mothers who agreed to participate in the study filled out an informed consent form. The form was composed of two parts: the research description and quotation of the relevant laws, which was retained by the mothers; the signature sheet expressing the consent for their participation in the study together with an agreement on data disclosure, which was retained by the first author.

The dyads were individually observed in their domestic settings during a book reading interaction. The instructions given to the mothers were as follows: “Look at this picture-book with your child and behave as you normally do when you read a picture-book at home.” The observer familiarized himself with each dyad before each observation and did not interfere in any way during the narrative interaction, in order to maintain ecological validity.

The picture-book (“*Frog, where are you?*”; Mayer, 1969) was about the adventures of a frog in the pond, and has been largely employed in previous research (e.g., Reilly et al., 2004). The instrument was considered to be appropriate for the present study, because it did not induce any particular way of telling the story and therefore allowed the mothers to exhibit their usual narrative style. The duration of the sessions was variable, ranging from 15 to 25 min. To assess maternal style, the narratives were audio-recorded, transcribed and then scored by two independent judges.

The coding scheme included three successive steps:

(1) In the first instance, the transcripts were divided into utterances – the minimal unit of analysis, formally defined as “an uninterrupted stream of language, which is distinguished from other utterances on the basis of lengthy pauses, grammatical structure, and changes in vocal intonation” (Longobardi et al., 2016a, p. 758). Each utterance was then classified as Narrative or Paradigmatic by two independent coders – inter-rater reliability (Cohen’s κ) was 0.83, and each disagreement was resolved through discussion with the first author. Maternal style was finally defined by the relative prevalence of narrative or paradigmatic utterances (more than 50%) on the total number of utterances produced during the narration, as in Tessler and Nelson (1994). **Table 1** reports examples of the eight narrative categories observed in the present study, out of the 18 originally proposed by Tessler and Nelson (1994). All other categories were excluded because they were not relevant for the present investigation.

(2) Then, the transcripts were coded according to the eight categories of mental lexicon used by Camaioni et al. (1998, modified; see **Table 2** for the coding scheme). Following these authors, two measures were computed: the overall proportion of internal state terms produced by each mother, out of the total number of words used during the interaction; and the relative frequency of each category, out of the total number of internal state terms produced during the interaction. Inter-rater reliability for coding each mental term in the eight categories was good (Cohen’s κ = 0.83).

(3) Lastly, for each internal state term produced by mothers, the two judges coded whether it referred to the mother, to the child, to the dyad, to the story characters (coded as ‘context’), or to others – identified as entities other than the mother–child couple and the characters described in the picture book.

**Table 1:** Coding scheme used to categorize maternal utterances (Tessler and Nelson, 1994; Rollo, 2003, Unpublished).

Style	Categories	Definition	Example
Narrative	(1) Describe activity	A depiction in basic, “surface” form of a behavior or occurrence taking place in view of mother and child.	*This frog is swimming in the pond.*
	(2) Autobiographical	A reference to something in the (usually shared) personal past; often a way of explaining by means of connecting the sight/activity/occurrence with something already experienced by the child.	*Where were we, not so long ago, when we saw something like this?*
	(3) Aesthetic/Affective	A depiction in aesthetic rather informative terms or expression of an emotion or attitude toward the thing observed.	*Those white flowers are beautiful, aren’t they? I love those! They’re my favorite!*
	(4) Interpretation	Utterance going beyond the basic information given about the feelings, intentions, possible future sequence of events or behavior relating to the person or object being observed.	*The frog is jumping on the stone because she’s afraid to drown*
Paradigmatic	(5) Describe category	Utterance labeling an object (or person) defining class it belongs to.	*What kind of animal is that? A bird, right!*
	(6) Basic knowledge	Often used for purpose of explaining something in the present situation referring to something child already know (“semantic” knowledge rather than “episodic” experience).	*Sure, you know that. Where do frogs live?*
	(7) Specific physical properties/size	Reference, often for purpose of focusing attention, to perceptual properties of the object.	*Look at these flowers, as they are great!*
	(8) Similarities/generalizations	Also used as a form of classification.	*This is the same kind as that one.*


**Table 2:** Coding scheme used to categorize the internal state words produced by mothers.

Type of internal states	Examples of internal state words used by mothers
Physiological	To be hungry, to be thirsty, to be sleepy.
Perceptual	To hear, to see, to look, to observe, to recognize, to be cold, to be hot, to feel ill.
Emotional positive	To enjoy, to be friends, to love, cheerful, happy, nice, satisfied.
Emotional negative	To be afraid, to become angry, to be sorry, to sorrow, unhappy, unpleasant, sad.
Volitional	To have intention of, to look for, to order, power ( = to be able of), to want, good.
Cognitive	To know, to understand, to remember, to forget, to dream, to think.
Communicative	To say, to tell, to call, to ask.
Moral	Duty (to be obliged to do), power ( = to have the permission), to forgive, good, bad.


## Results

### Mothers’ Communicative Styles

The first aim of this study was to identify two different maternal styles used during the picture-book reading interactions. Following the procedure outlined in the “Materials and Methods” section, 14 mothers out of 30 (46.7%) were classified as Narrative, while the remaining 16 mothers were classified as Paradigmatic (53.3%). When analyzed with respect to children’s age groups, the two maternal styles showed a clear developmental trend that coincided with the increase in children’s age. Mothers of 4.05-year-old children were more Narrative than Paradigmatic (90% vs. 10%); in contrast, the mothers of older children resulted to be more Paradigmatic than Narrative [70% vs. 30% and 80% vs. 20%, respectively; χ^2^_(2)_ = 11.52, *p* < 0.001]. Overall, these results confirm the existence of the two maternal styles observed in the earlier study (Tessler and Nelson, 1994), but additionally indicate that mothers adjust their narrative style depending on children’s age.

Interestingly, there were no significant differences [*F*_(2,27)_ = 0.050; *p* = 0.951] in the total number of utterances produced by mothers: *M* = 26.60 (age 4.05), *M* = 27.70 (age 5.05), and *M* = 24.60 (age 5.11). The latter finding suggests that differences in the narrative styles were not due to differences in maternal verbosity.

### Mothers’ Mental State Language

Regarding the mothers’ use of MSL during the picture-book reading, the overall percentages of internal state terms, out of the total of words produced by mothers, ranged from a minimum of 1.37 to a maximum of 11.39 (*M* = 7.71; *SD* = 2.53). Considering the different categories of internal state language, **Table 3** shows that emotional (positive), perceptual, and communicative terms were used more frequently than the other mental state terms.

**Table 3:** Descriptive statistics for mothers’ categories of MSL.

Terms of mothers’ MSL	% *M*	*SD*
		
Physiological	0.46	1.75
Perceptual	19.13	11.87
Emotional positive	18.76	14.10
Emotional negative	5.25	9.51
Volitional	14.35	19.55
Cognitive	17.82	11.70
Communicative	19.37	11.33
Moral	3.94	8.32


**FIGURE 1:**
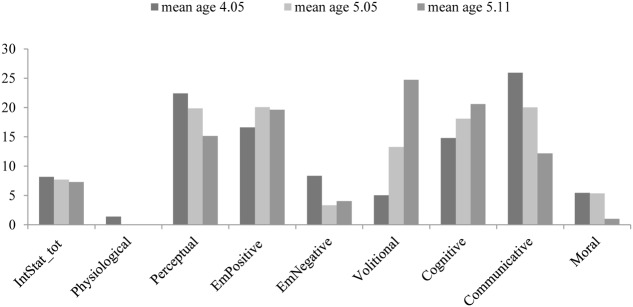
Mean percentages of mental state terms used by mothers, as a function of category (physiological, perceptual, emotional positive, emotional negative, volitional, cognitive, communicative, and moral) and children’s age. IntStat_tot = total percentages of mental state words.

In **Figure 1**, we can observe the types of internal state words used by mothers with their children. It can be noted that the mothers of younger children used a wider variety of inner state words referring to perception and emotion, while the mothers of older children used more inner state words referring to cognitive states or volition.

To formally verify differences in the mothers’ use of MSL categories as a function of children’s age, we performed a MANOVA (multivariate analysis of variance) that has showed a significant difference in the use of communicative terms [*F*_(2,27)_ = 4.498; *p* = 0.021; η^2^ = 0.44] with a decreasing trend: mothers of younger children utilized more communicative terms (*M* = 25.93, *SD* = 11.70) than mother of the 5.05-year-old children (*M* = 20.03, *SD* = 10.19) and the 5.11-year-old children (*M* = 12.17, *SD* = 8.15). *Post hoc* comparisons using Bonferroni test indicated that the use of communicative terms was significantly lower in the mother of older children (5.11-year-old) than in the mother of younger children (4.05-year-old) (*p* = 0.016).

A marginally significant increase was instead noted in the use of volitional terms [*F*_(2,27)_ = 2,788; *p* = 0.080]. Mothers of 5.11-year-old children utilized more volitional terms (*M* = 24.73, *SD* = 29.31) than mothers of the 5.05-year-old children (*M* = 13.26, *SD* = 10.60) and the 4.05-year-old children (*M* = 5.05, *SD* = 6.48).

**FIGURE 2:**
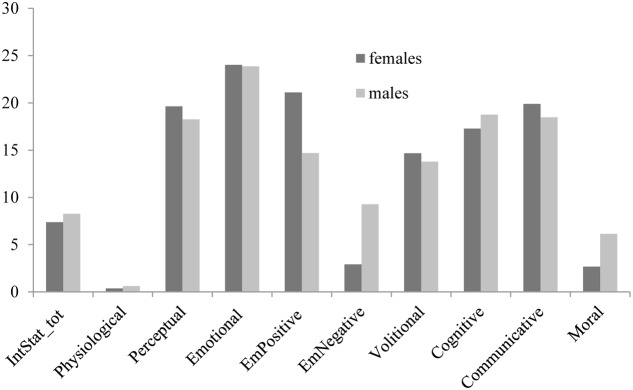
Mean percentages of mental state terms used by mothers, as a function of category (physiological, perceptual, emotional positive, emotional negative, volitional, cognitive, communicative, and moral) and children’s gender. IntStat_tot = total percentages of mental state words.

With regards to children’s gender, no significant difference was found: mothers used internal state terms with girls and boys similarly. However, at a descriptive level (see **Figure 2**), it is interesting to note that mothers addressed emotional positive states more frequently to girls than to boys [*F*_(1,28)_ = 1.313; *p* = 0.262], whereas emotional negative states were directed more frequently to boys than to girls [*F*_(1,28)_ = 3.065; *p* = 0.092].

### Maternal Communicative Styles and Mental State Language

**FIGURE 3:**
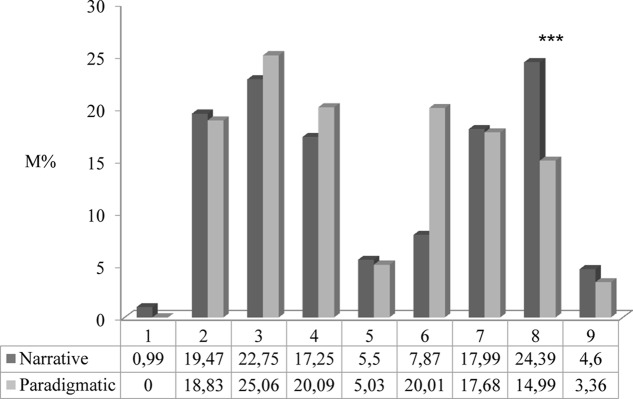
Mean percentages of mental state terms used by mothers, as a function of category (physiological, perceptual, emotional positive, emotional negative, volitional, cognitive, communicative, and moral) and narrative style. 1 = Physiological words; 2 = Perceptual words; 3 = Emotional words (Emotional positive + Emotional negative); 4 = Emotional positive words; 5 = Emotional negative words; 6 = Volitional words; 7 = Cognitive words; 8 = Communicative words; 9 = Moral words.

A primary aim of this research was to verify whether mothers classified as Narrative or Paradigmatic differed with respect to the use of psychological lexicon. A MANOVA carried out on the eight categories of mental state terms found only one significant difference between maternal style groups [*F*_(1,28)_ = 5.872; *p* = 0.022; η^2^ = 0.54]: Narrative mothers used communicative terms more frequently than Paradigmatic mothers (see **Figure 3**). In addition, looking at **Figure 3** it can be noted that there was a marginally significant difference for the volitional category [*F*_(1,28)_ = 3.455, *p* = 0.074], with Paradigmatic mothers using these terms more frequently than Narrative mothers. In contrast, Narrative mothers tended to use physiological terms more frequently than Paradigmatic mothers [*F*_(1,28)_ = 3.069, *p* = 0.091].

### Referents of Maternal Mental State Language

The last aim of the present study was to verify whether Narrative and Paradigmatic mothers differed in the way in which they referred mental state terms to themselves, to the child, to the dyad, to characters of the picture book (context) or to others (persons not described the book).

Overall, internal state words were referred preferably to the context (*M* = 41.33) or to the child (*M* = 30.06), while references to others (*M* = 13.28), to the mother (*M* = 9.71), or to the dyad (*M* = 4.98) were relatively rare. A MANOVA carried out on the referents of mental state terms revealed no significant differences between Narrative and Paradigmatic mothers [for example, References to the child: *F*_(1,28)_ = 1.505; *p* = 0.23; References to the context: *F*_(1,28)_ = 1.459; *p* = 0.24]. Interestingly, however, Pearson’s correlations revealed that context references were negatively and significantly related to references to the mother (*r* = -0.53), to the child (*r* = -0.67) and to others (*r* = -0.44) (all *p*s < 0.01). These results suggest that the more the mothers referred mental state terms to the book characters, the less they referred them to themselves, to the children or to other persons out of the dyad (for example, their relatives).

As a last point, we analyzed possible differences in the references of maternal MSL as a function of children’s age. The MANOVA results, reported in **Table 4**, show that the mothers of younger children referred their MSL to the context more frequently than the mothers of older children. *Post hoc* comparisons with the Bonferroni correction showed that the mothers of 4.05-year-old children referred mental state terms to the context more often the mothers of 5.05-year-old children (*p* = 0.031).

**Table 4:** Mean percentages of mental state terms referred to mother, child, mother and child (dyad), context and others, as a function of children’s age.

References of mothers’ MSL	Children’s age in years			*F*_(2,27)_	*P*
			
	4.05 (*N* = 10)	5.05 (*N* = 10)	5.11 (*N* = 10)		
Mother	4.5 (6.37)	13.22 (14.88)	11.41 (13.73)	1.3	0.29
Child	24.45 (13.59)	30.35 (16.35)	35.39 (16.61)	1.19	0.32
Mother and child	2.00 (2.92)	9.68 (11.46)	3.22 (4.96)	3.11	0.061
Context	57.31 (17.38)	30.22 (21.35)	36.45 (26.24)	4.02	0.030
Other	11.72 (8.23)	14.62 (11.29)	13.51 (12.87)	0.235	0.79


*Statistics and *p*-values (MANOVA) are reported in the last two columns.*

## Discussion

Narratives provide a coherent structure for recounting the past to others, but also for representing the past to ourselves (Rollo and Benelli, 2003). Since language is the medium through which children learn the canonical narrative structures (Bruner, 1991, 1996), it is also the primary means through which adults “socialize” children (Fivush, 2014). In this context, mother–child conversations are essential instruments through which children learn the cultural norms that guide adult’s behaviors. As Fivush et al. (2006) wrote: “Children’s lives are organized such that the activities in which children are encouraged to engage are those that the culture deems important, and it is through participation in culturally mediated, socially structured activities that children learn the skills necessary to become competent members of their culture. With time, as children engage in adult-guided activities, they internalize the skills initially displayed by others and add them to their own intrapersonal repertoire” (Fivush et al., 2006, p. 1,568). It is then important to thoroughly investigate this continuous and circular process where adults narrate their own experiences and children learn to build their personal representations: how is the adults’ narrative toward children organized?

Parents and their children spend many time in joint picture-book reading. This activity conforms to the turn-taking structure of conversation and occurs in a repetitive sequence formerly described by Ninio and Bruner (1978). Furthermore, it is from the frequent exposure to this format that children acquire the capacity to create stories. Since picture-book reading is characterized by a unique three-way intersection of narration, face-to-face interaction and self-reference, it provides an optimal setting for exploring the processes of self-construction (Lonigan and Whitehurst, 1998). Indeed, numerous studies have investigated the relationship between maternal mental lexicon during picture-book reading and their children’s ToM (Ontai and Thompson, 2008). These studies examined both the variations in maternal styles and the consequences on children’s understanding of mind. Although the results have highlighted a close association between maternal mental lexicon and the development of children’s social comprehension, it is not yet clear which aspects of maternal language are essential to the establishment of this relation.

Within this broad context, the present study focused on maternal talk to 4- to 6-year-old children, to investigate: (a) whether narrative styles changed as a function of children’s age; and (b) whether different narrative styles were associated to quantitative differences in the use of mental state terms, including their referents. First, we wondered whether it was possible to discriminate between mothers who utilized Narrative versus Paradigmatic styles. Within our relatively small sample, we found mothers who, during the task of picture-book reading, were highly consistent in referring to the autobiographical experiences of their children and the mental states of the book characters, and mothers who were primarily focused on specifying the properties of objects and their relations. In this respect, the results of our study confirm the distinctions illustrated in the literature between elaborative versus repetitive styles (Fivush and Fromhoff, 1988), narrative versus paradigmatic styles (Tessler, 1986, Unpublished; Tessler and Nelson, 1994), or expressive versus referential styles (Nelson, 1973). Clearly, these categories should be considered as the extremes of a continuum, since all the mothers produced utterances of both types. More interestingly, we found that the mothers’ use of narrative styles varied as a function of children’s age, although the direction of this difference did not replicate the results reported by Reese et al. (1993). In facts, our data indicate that the mothers of 4-year-old children used primarily an expressive/narrative speech, more centered on interactions and shared experiences, whereas the mothers of 5- and 6-year-old children were more likely to adopt a referential/paradigmatic speech, more focused on objects. This modification parallels what happens during early child development. As children develop and refine their interaction skills, they move from a narrative to a paradigmatic way of systematizing the reality around them (Rollo, 2003, Unpublished); likewise, mothers change their way of conversing with their children.

The second aim of the present study was to identify the internal state words used by mothers during the picture-book reading. To this purpose, the transcripts were coded using a scheme illustrated by Camaioni et al. (1998), which differentiates between eight different MSL categories (physiological, perceptual, emotional negative, emotional positive, cognitive, volitional, communicative, and moral). Confirming previous studies that examined the MSL addressed to 24-month-old children (Longobardi et al., 2016a), we found that perceptual terms were used with the highest frequency, followed by emotional positive, cognitive, and volitional terms. Importantly, communicative terms were produced with the same frequency of perceptual terms, suggesting that their use might be particularly relevant for the creation of a narrative frame in which children begin to build mature representations of themselves and other people (Nelson and Fivush, 2004). Indeed, the terms “to say,” “to tell,” and “to call” were frequently used by the mothers of 4.05-year-old children.

Statistical analyses revealed that the mothers’ use of communicative terms varied as a function of children’s age, being more frequent in the mothers of younger than older children. No other difference reached the significance level, although the general trend was well in line with the available literature. For example, the use of perceptual terms was quite frequent with 4-year-old children but decreased sharply in the mothers of older children; in contrast, the use of cognitive and volitional terms was relatively limited during the interactions with the youngest group of children, but exhibited a strong increase in the mothers of 5- and 6-year-old children. These results nicely mirror the results observed in previous studies which focused on children’s production and comprehension of internal state terms (Bretherton and Beeghly, 1982; Shatz et al., 1983; Tardif and Wellman, 2000; Wellman and Liu, 2004; Kristen et al., 2012; Longobardi et al., 2014), and therefore confirm the causal role of maternal language (Meins et al., 2002; Ruffman et al., 2002; de Rosnay et al., 2004; Slaughter et al., 2007; Ornaghi et al., 2011). Furthermore, the mothers in our sample used emotional terms more frequently with girls than with boys – a finding which has been already observed in the literature (Lagattuta and Wellman, 2002).

A central aim of our research was to verify whether mothers classified as Paradigmatic or Narrative differed with respect to the use of MSL. In contrast with our original hypotheses, the present findings suggest that conversational styles and MSL are rather independent: indeed, Narrative and Paradigmatic mothers used the eight categories of MSL to the same degree, with one noteworthy exception: that Narrative mothers produced communicative terms more frequently than Paradigmatic mothers. This is interesting because the frequent use of verbs such as “tell” and “say” might facilitate children’s understanding of tensed complements, which in turn “is a precursor and possible prerequisite of successful false-belief performance” (e.g., de Villiers and Pyers, 2002, p. 1,037). In agreement, studies have suggested that mothers’ production of communicative (and more generally, cognitive) verbs might be closely related to children’s understanding of false belief, representational change and the appearance-reality distinction (Le Sourn-Bissaoui and Deleau, 2001; Adrián et al., 2007).

Finally, we wanted to verify whether mothers referred internal state words to “others” (including their children and the book characters) more than to themselves. In this respect, correlational analyses showed that the more the mothers referred mental state lexicon to the book characters, the less they referred it to the children or to themselves, indicating a dialog between two different entities: the fictional characters of the story and real persons – the mothers and their children. Assuming that during the book-reading interaction there is a “match” among these three components (Reese et al., 2003; Fletcher and Reese, 2005), the social interaction established between these elements seems to move from the book and its “concrete characters” to the children and the mothers. This was confirmed by the results of the MANOVA, which showed that the attributions of MSL to the book characters were predominant in 4.05-year-old children; this suggests that mothers can tailor their mentalistic input to the abilities of younger children: in facts, the book characters provide a concrete anchoring to verbally expressed mentalistic content. In other words, the mothers of younger children use the book to objectify an internal reality, which later will be directly attributed to the child. Moreover, they adapt the language produced in shared narrative contexts to the age of their children, in order to guide them toward a gradual awareness of their inner world (Rollo, 2003, Unpublished).

## Conclusion and Future Directions

In summary, the present study examined the characteristics of maternal talk during narrative interactions with their children, with a particular focus on the different use of MSL by Narrative and Paradigmatic mothers. We learned that: (a) maternal narrative styles changed as a function of children’s age, such that the mothers of 4-year-old children were more Narrative than Paradigmatic, whereas the mothers of 5- and 6-year-old children exhibited the opposite pattern (i.e., they were more Paradigmatic than Narrative); (b) the use of communicative terms decreased with children’s age, and was more frequent in Narrative than in Paradigmatic mothers; and (c) mothers adapted the referents of their MSL to the children’s age, such that internal state terms were first referred to the book characters, then to the child themselves, and lastly to the dyad.

These conclusions should be evaluated with caution, because the present study has several limitations. First, the sample size was relatively small, and this factor limited the possibility to observe significant differences between the three groups of children. A second limitation was that we focused on mothers’ speech, and therefore no data about children’s language was collected. This means that we could not assess whether changes in maternal MSL were partially due to simultaneous changes in the internal and non-internal language produced by children. Finally, no attempt was made to investigate children’s performance in false belief tasks, although previous research indicates a close link between children’s ToM and mothers’ MSL (Furrow et al., 1992; Jenkins et al., 2003; Rudek and Haden, 2005; Symons et al., 2006; Pearson and Pillow, 2016). Even with these limitations in mind, we believe that our results may represent a starting point for future research.

Longitudinal studies should be conducted to delineate any parallel change in children’s social and emotional development. Moreover, interventions can be planned in cases in which the narrative interactions between mothers and children are limited in frequency or dysfunctional (Tompkins and Farrar, 2011). In this respect, the use of language intervention practices is now well established in a variety of conditions, including children with language impairments (Cirrin and Gillam, 2008), children coming from economically disadvantaged families (Cain et al., 2005), and bilingual/immigrant children (Pirchio et al., 2015, 2017). This growing body of research has begun to document the fact that training mothers to talk elaboratively about past events can have positive effects on children’s understanding of mind (Peterson et al., 1999; Reese and Newcombe, 2007; Reese et al., 2010a,b; Taumoepeau and Reese, 2013). More generally, interventions that induced caregivers, teachers, and educators to increase the frequency of book-reading interactions have reported significant benefits to children’s oral language and their emergent psychological lexicon (Whitehurst et al., 1999; Aram and Biron, 2004; Aram et al., 2013; Grazzani et al., 2016). The present study adds to this literature by suggesting that, in addition to enhancing the overall quantity of elaborations produced by caregivers, training interventions should also focus on fostering the use of specific categories of mental state terms (communicative, volitional, and cognitive) and increasing the references to agents other than the book characters.

## Ethics Statement

This study was carried out in accordance with the recommendations of AIP guidelines, with written informed consent from all subjects. All subjects gave written informed consent in accordance with the Declaration of Helsinki. The protocol was approved by the local ethical committee.

## Author Contributions

All authors listed have made a substantial, direct and intellectual contribution to the work, and approved it for publication.

## Conflict of Interest Statement

The authors declare that the research was conducted in the absence of any commercial or financial relationships that could be construed as a potential conflict of interest. The reviewer SP declared a shared affiliation, with no collaboration, with the authors PS and EL to the handling Editor.
